# Effect of Al_2_O_3_ Passive Layer on Stability and Doping of MoS_2_ Field-Effect Transistor (FET) Biosensors

**DOI:** 10.3390/bios11120514

**Published:** 2021-12-13

**Authors:** Tung Pham, Ying Chen, Jhoann Lopez, Mei Yang, Thien-Toan Tran, Ashok Mulchandani

**Affiliations:** 1 Department of Chemical and Environmental Engineering, University of California Riverside, Riverside, CA 92521, USA; tpham052@ucr.edu (T.P.); ychen751@ucr.edu (Y.C.); jlope086@ucr.edu (J.L.); 2 Key Laboratory of Biorheological Science and Technology Ministry of Education, College of Bioengineering, Chongqing University, Chongqing 400044, China; yangmei@cqu.edu.cn; 3 Department of Bioengineering, University of California Riverside, Riverside, CA 92521, USA; ttran6@nd.edu; 4 Center for Environmental Research and Technology (CE-CERT), University of California Riverside, Riverside, CA 92507, USA

**Keywords:** MoS_2_, metal oxide, biosensor, chemiresistive, field-effect transistor, chemiresistor

## Abstract

Molybdenum disulfide (MoS_2_) features a band gap of 1.3 eV (indirect) to 1.9 eV (direct). This tunable band gap renders MoS_2_ a suitable conducting channel for field-effect transistors (FETs). In addition, the highly sensitive surface potential in MoS_2_ layers allows the feasibility of FET applications in biosensors, where direct immobilization and detection of biological molecules are conducted in wet conditions. In this work, we report, for the first time, the degradation of chemical vapor deposition (CVD) grown MoS_2_ FET-based sensors in the presence of phosphate buffer and water, which caused false positive response in detection. We conclude the degradation was originated by physical delamination of MoS_2_ thin films from the SiO_2_ substrate. The problem was alleviated by coating the sensors with a 30 nm thick aluminum oxide (Al_2_O_3_) layer using atomic layer deposition technique (ALD). This passive oxide thin film not only acted as a protecting layer against the device degradation but also induced a strong n-doping onto MoS_2_, which permitted a facile method of detection in MoS_2_ FET-based sensors using a low-power mode chemiresistive I-V measurement at zero gate voltage (V_gate_ = 0 V). Additionally, the oxide layer provided available sites for facile functionalization with bioreceptors. As immunoreaction plays a key role in clinical diagnosis and environmental analysis, our work presented a promising application using such enhanced Al_2_O_3_-coated MoS_2_ chemiresistive biosensors for detection of HIgG with high sensitivity and selectivity. The biosensor was successfully applied to detect HIgG in artificial urine, a complex matrix containing organics and salts.

## 1. Introduction

Due to a high surface-area-to-volume ratio and up to centimeter-scale lateral dimension, 2D materials are ideal for integration with current nanofabrication technologies and highly promising transduction materials in sensor technologies that rely primarily on surface interaction [1]. A prominent example of functional 2D nanomaterials is graphene, which was the first 2D material isolated from its bulk structure in 2004 and has been extensively investigated. Despite its extraordinary properties, such as high optical transparency, thermal conductivity up to 3000 W m^−1^ K^−1^, high charge carrier concentration, and mobility [2], the lack of an energy gap between the conduction and valence bands limits its application in electronic devices. Attempts to address this deficiency have led to several approaches to engineer a bandgap in graphene. However, most of the successful methods require lengthy and complicated processes of fabrication using e-beam lithography or unstable doping treatment with harsh chemicals [3,4]. These intensive processes often lead to a significant decrease in carrier mobility and an increase in the number of defects in graphene. This challenge has encouraged researchers to find alternative 2D materials, and transition metal dichalcogenides (TMDCs) have become an emerging field of research. Unlike graphene, TMDCs possess a direct bandgap in the range of 0.2 to 3 eV in their single-layer (SL) structure [5]. This intrinsic band gap suggests that TMDCs are suitable for electronic/transistor applications. Molybdenum disulfide (MoS_2_) is one of the most studied TMDC compounds. It consists of one layer of molybdenum atoms in between two layers of sulfur atoms. SL-MoS_2_ exhibits a direct bandgap of 1.87 eV, which transitions to an indirect bandgap as the number of layers increase. Therefore, a field-effect transistor (FET) using SL-MoS_2_ as a semiconducting channel is expected to have a high on/off current ratio of up to 10^8^ and excellent subthreshold swing of 74 mV/decade [6]. In addition, due to a planar structure confined to a few angstroms thickness, MoS_2_ is ultrasensitive to chemical and electrostatic perturbations at the surface. These advantages make MoS_2_ a great choice for transduction or sensing material in FET-based biosensors.

In the field of biosensor research, label-free affinity-based biosensing is a desirable attribute for biosensor systems due to its simplicity. Amongst the different transducers used for label-free affinity biosensors, FETs and chemiresistors based on 1D and 2D nanomaterials are receiving a great deal of attention because of their high sensitivity, rapid response, low power consumption, viability for miniaturization, scalability, and integration on a chip. While methods of electrical detection either with an applied gate (FET characteristic measurement) or without an applied gate (chemiresistive measurement) have been demonstrated for biosensors using semi-metallic graphene, semiconducting graphene derivatives and carbon nanotubes [7,8,9,10], MoS_2_-based electrical biosensors heavily rely on FET techniques, in which a source-drain current (I_ds_) is measured at a constant bias while varying the applied gate voltage to modulate the I_ds_. This mandatory gate voltage serves as an amplifier of the small current by enhancing the carrier mobility, originated by the nature of low charge mobility in semiconducting MoS_2_ and high Schottky barrier at the interface of MoS_2_/metal contact [11,12]. In addition, because the applied back-gating effect plays a key role in FET-based measurement, it requires a reliable and direct physical contact between MoS_2_ (the conducting channel) and the dielectric substrate. Nonuniform and unstable contact at the materials interface can result in unreliable electrical modulation via gating effects, further leading to degradation of signal integrity and reliability during sensing.

A necessary step in the fabrication of a label-free MoS_2_-based biosensors is the immobilization of a biomolecular receptor to the surface of MoS_2_ to achieve biospecific responses. This biofunctionalization step is achievable via covalent and non-covalent conjugation strategies, such as hydrophobic interaction, silanization, and sulfide/disulfide bonding [13,14,15]. However, these methods either employ weak van der Waals bonds or significantly rely on the number of defects in MoS_2_, which mainly comprises sulfur vacancies, to create such attachments. As a result, MoS_2_-based biosensors are commonly fabricated using MoS_2_ synthesized via chemical/physical exfoliation or hydrothermal reaction. MoS_2_ synthesized via these methods produce a great number of defects permitting the direct functionalization of biomolecular receptors. On the other hand, biosensing applications using MoS_2_ grown by chemical vapor deposition (CVD) are relatively limited due to an inadequate number of the defect sites in CVD-grown MoS_2_ (CVD-MoS_2_) crystal lattice. Despite this hinderance, CVD-MoS_2_ exhibits a higher crystallinity and is amenable to mass production. For these reasons, CVD-MoS_2_ deserves more attention in research for biological and chemical sensing applications.

In this work, we report a systematic investigation to identify causes of signal instability of CVD-MoS_2_ FET-based biosensors and develop a strategy to mitigate the effects of physical degradation of the device and enhance the overall device stability. Specifically, we observed that CVD-MoS_2_-based biosensors degraded, indicated by dramatic change in their electrical outputs during mandatory incubation steps of the biofunctionalization process in 10 mM phosphate buffer (pH = 7.4) and deionized water (pH = 7). We conclude the observed continual shifts in device FET characteristics was attributed to physical delamination of MoS_2_ film from Si/SiO_2_ substrate, which decreased the gating effect and negated the usage of FET characteristic transfer curve as a mean for detection. This issue was mitigated by stabilizing the device with a 30 nm thick Al_2_O_3_ coating. In addition, the oxide layer provided available sites for antibody functionalization via surface chemical functionalization with (3-aminopropyl) triethoxysilane (APTES) and glutaraldehyde. Such sensors demonstrated a great performance to detect HIgG with high sensitivity and a limit of detection of 83 ng/mL.

## 2. Experimental Section

### 2.1. Materials and Agents

Si wafer with a 300 nm thermal grown oxide layer were purchased from Ultrasil (Hayward, CA, USA). Sulfur powder, molybdenum trioxide (MoO_3_), and polystyrene (PS) were purchased from Sigma-Aldrich (St. Louis, MO, USA). Monoclonal anti-human immunoglobulin G (HIgG) antibody, HIgG protein, bovine serum albumin (BSA), and human serum albumin (HSA) were purchased from Sigma-Aldrich Corp. (St. Louis, MO, USA). All organic solvents, salts, and components of artificial urine were purchased from Fisher Scientific (Hampton, NH, USA).

### 2.2. Synthesis of MoS_2_

MoS_2_ was synthesized via CVD method with sulfur and MoO_3_ as the precursors and Si/SiO_2_ as the substrate. A total of 0.01 g of MoO_3_ was centered in a ceramic boat with the Si/SiO_2_ substrate positioned on top of the MoO_3_ source. The growth took place at the center of a two-zone 1-in quartz tube furnace at 650 °C while the tube was saturated with sulfur vapor by heating 0.1 g of sulfur at 170 °C and using a nitrogen carrier gas at a flowrate of 50 sccm. The temperature was maintained at 650 °C for the complete reaction and the entire system was naturally cooled down to room temperature by removing the tube from the furnace.

### 2.3. Fabrication of FET Devices

First, 3% PS in toluene was spin-coated over the as-grown MoS_2_ film, and the PS coated MoS_2_ film was then baked at 60 °C for 1 min. The PS/MoS_2_ film was isolated from the substrate via simple wet etching of Si/SiO_2_ in 1 M KOH solution. The isolated film was washed several times with deionized (DI) water and transferred on a pre-cleaned Si/SiO_2_ substrate. The film was dried in ambient air and annealed at 80 °C for 15 min to increase the contact between MoS_2_ and Si/SiO_2_ substrate. PS layer was removed in toluene at room temperature.

Buffered oxide etch (BOE) 6:1 (Sigma-Aldrich, St. Louis, MO, USA) was used for etching the back side of the wafer, and a 10 nm/100 nm of Cr/Au was deposited on the silicon side of the Si/SiO_2_ as a back-gate. The source and drain terminals with a gap size of 10 µm × 10 µm were patterned using conventional photolithography followed by electron-beam evaporation of 10 nm/100 nm of Cr/Au.

### 2.4. Atomic Layer Deposition (ALD) of Aluminum Oxide Layer

Aluminum oxide layers were deposited using Cambridge Nanotech Savannah 100 from Veeco Instruments, Inc. (Plainview, NY, USA) at 250 °C. Trimethylaluminum (TMA) and water, as precursors, were pulsed in the system periodically for 0.03 s. The thickness of the oxide layer was varied by changing the number of cycles, estimated at about 1 Å/cycle. Aluminum oxide of 10 nm and 30 nm thickness were grown using 100 and 300 cycles.

### 2.5. Materials Characterization

All optical images were taken using a Hirox KH-7700 digital microscope from Seika Machinery, Inc. (Los Angeles, CA, USA). Al_2_O_3_ thickness was measured by Jobin Yvon UVISEL model M200 Ellipsometer from Horiba (Kyoto, Japan). Both Raman spectra and PL measurements were collected by Horiba LabRam HR (Kyoto, Japan) using a green laser (λ = 532 nm) and 100× objective (NA = 0.9). A minimal power of 5 mW was used to avoid local heating and possible damage to the materials. SEM images were taken by a Zeiss STEM Gemini 1540xb (Oberkochen, Germany). 

FET characteristics curves were obtained by Keithley 2636 source meter from Tektronics (Beaverton, OR, USA). A constant V_ds_ = 5 V and V_ds_ = 0.1 V were applied while sweeping the back-gate voltage from 0 V to 40 V.

### 2.6. Biofunctionalization and Biosensing of HIgG

The Al_2_O_3_ passivated MoS_2_-based device was immersed in 5 mL of 20% ammonium hydroxide for 30 min at room temperature (RT) ensuring that -OH groups were sufficiently activated on the surface and improving the formation of the homogeneous layer. The device was then carefully rinsed with DI water and dried with nitrogen gas. Subsequently, the device was incubated in 1 mL of APTES for 1 h and washed thoroughly with ethanol to form a self-assembled monolayer providing amino groups for covalent attachment of anti-human immunoglobulin G (HIgG) antibody, which interacted selectively with HIgG. APTES amino groups were, first, activated by placing the device in 4 mL of 25% glutaraldehyde in DI water for 2 h to form a Schiff base between -NH_2_ of APTES and -CHO of glutaraldehyde at RT. This was followed by overnight incubation at 4 °C with 20 μL of 50 µg/mL anti-HIgG in phosphate buffer (PB) to form a Schiff base between the 2nd -CHO of glutaraldehyde and -NH_2_ of anti-HIgG. Lastly, the device was reacted with 20 μL of 10% ethanolamine to quench unreacted -CHO groups of glutaraldehyde.

The biofunctionalized devices were applied to detect HIgG antigen in both PB and AU solution. A 30 µL customized PMMA cell was employed for the detection. A pair of inlet/outlet tubes was connected to the test cell and used to deliver a series of reagent fluids, including PB (10 mM, pH 7.4), AU (pH 7.4) solution, and analyte solutions of HIgG. A volume of 200 µL of solution was injected into the sensor test cell for each run while the dynamic change in electrical resistance of the sensor was monitored continuously at an applied source-drain voltage of 1 V (with no gate voltage), using a Keithley source meter.

## 3. Results and Discussion

**Figure 1:**
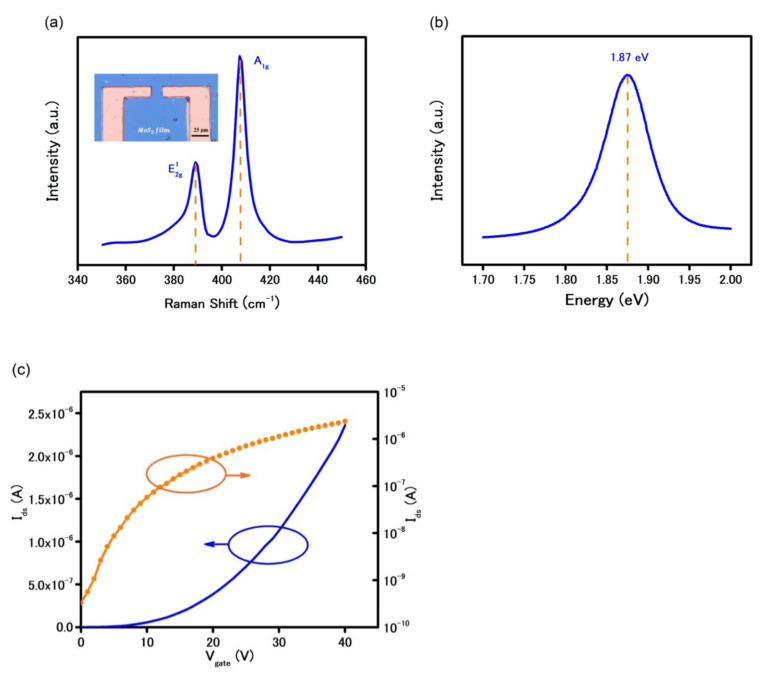
(**a**) Spectroscopic properties of CVD-MoS_2_: Raman spectrum and optical image of MoS_2_ device (inset). (**b**) Photoluminescence of CVD-MoS_2_ showing a direct band gap of 1.87 eV. (**c**) Field-effect transistor characteristics curve using MoS_2_ as the conducting channel (V_ds_ = 5 V).

The optical image in Figure 1a (inset) shows the MoS_2_ synthesized by CVD was a continuous film that remained a continuous film after the transfer. The similarity of color contrast across the film illustrates the growth of MoS_2_ was uniform and the transfer was residue-free.

The film thickness and electrical bandgap of the synthesized MoS_2_ were confirmed by different techniques such as Raman spectroscopy, photoluminescence (PL) under a green laser with spot size ≈ 1 µm. The presence of two signature peaks of E_2g_ at 385 cm^−1^ and A_1g_ at 404 cm^−1^ in Figure 1a confirms the successful growth of MoS_2_. In addition, the difference in wavenumber of 19 cm^−1^ between the two peaks indicates the as-grown MoS_2_ was single-layer [16]. Due to the intrinsic direct bandgap in SL MoS_2_ structure, the photoluminescence spectrum in Figure 1b shows a significantly high peak at 1.87 eV.

A back-gated FET using CVD-MoS_2_ as the semiconducting channel was prepared using conventional photolithography process. As shown in Figure 1c, the FET characteristics curve shows a typical n-type behavior with a high on/off current ratio of 10^4^ in ambient condition. Under the applied positive back-gate voltage, the device was turned on, i.e., dramatic increase in current due to the accumulation of electrons in MoS_2_. The field-effect mobility was calculated to be 20.5 cm^2^V^−1^s^−1^, using the linear FET characteristics reported elsewhere [17]. This low charge mobility is mainly caused by charge trapping at the interface between the SiO_2_ substrate and MoS_2_ [18,19] and high contact resistance at metal/MoS_2_ interface [11]. Because of such challenges, electrical measurement methods in MoS_2_-based devices are limited to a FET characteristics curve with applied gate voltage [13,20,21].

**Figure 2:**
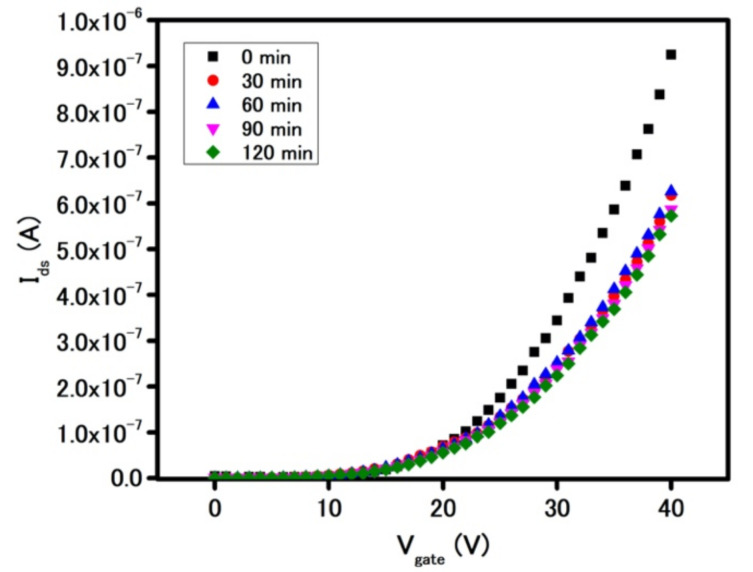
Stability of MoS_2_ device in ambient air.

Due to the significant role of the FET measurement in MoS_2_-based sensors, the stability of as-fabricated FET devices becomes critical. A stability test was carried out initially in ambient air and later in both biocompatible media of phosphate buffer (PB, pH = 7.4) and DI water (pH = 7). As shown in Figure 2, the FET characteristics curve experienced a measurable decrease in current after 30 min in ambient air, which was due to the interaction between MoS_2_ film and oxidizing gas molecules in air [22]. However, after 30 min and for an additional 90 min under the same condition, the FET characteristics curve remained unchanged, which indicates physical and chemical pseudo-steady-state in the device.

**Figure 3:**
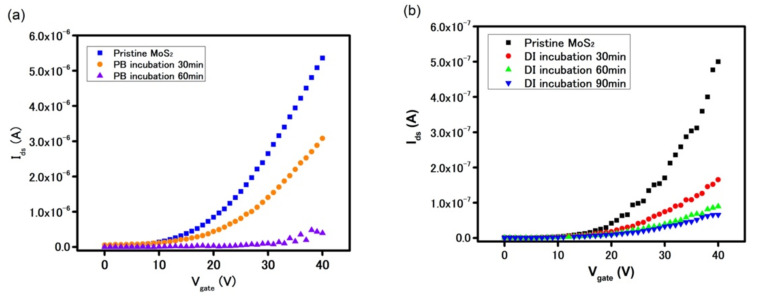
FET characteristics curve of MoS_2_-based device in (**a**) PB (pH = 7.4) and (**b**) DI water (pH = 7).

On the other hand, upon incubation in PB for 30 min by drop casting of 20 µL of PB onto the sensing area, the device showed a significant decrease in overall I_ds_ current across the entire range of applied gate voltages as seen in Figure 3a. In fact, the current at the gate voltage of 40 V dropped by 33% after 30 min of incubation, as compared to further 1.6–5% drop after additional 90 min. Similar deterioration in electrical behavior was observed when the device was incubated in DI water (Figure 3b). Thus, in considering the similar magnitudes of diminution of I_ds_ current for the device in the ionic PB environment and deionized water environment, this suggests the presence of water molecules as the major contributing factor for the observed electrical behavior of the device, instead of the buffer salt ions. Three possible mechanisms for how water could diminish the performance of a FET MoS_2_-based device were considered:

1. Water altered the chemical properties of MoS_2_ during the incubation via -O or -OH bonding;
2. Water intercalated between gold electrodes and MoS_2_ and increased the contact resistance;
3. Water intercalated between MoS_2_ and the substrate and decreased the gating effect on the MoS_2_ channel.

Raman spectrometry was employed to examine the properties of MoS_2_ after 30 min incubation in DI water [23]. As illustrated in Figure S1, the absolute intensity of the two signature peaks of MoS_2_ remained unaffected after the incubation, indicating there was no oxidation taking place. As a result, the mechanism (1) of water induced chemical effect on MoS_2_ film was disregarded.

To determine if the phenomenon of water intercalation between MoS_2_ and gold electrodes was occurring, controlled experiments were conducted where MoS_2_-electrode interfacial regions of the FET device were physically isolated (via passivation with photoresist) from the immediate buffer environment. A thin layer of positive photoresist S1813 was spin-coated on top of the device, and a small photoresist area of 10 μm × 10 μm at the contact between MoS_2_ and gold electrodes was removed using photolithographic patterning. An optical image of such a schematic can be found in Figure S2a. This polymeric layer behaved as a hydrophobic layer that prevented water from intercalating between the gold contacts and MoS_2_ but still allowed the possibility of water inserting between MoS_2_ film and Si/SiO_2_ substrate due to the open window of 10 μm × 10 μm. Figure S2b shows the resulting FET characteristic curves of pre-photoresist, post-photoresist, and after device incubation from 30 to 120 min in DI water. As a protecting layer, S1813 film minimized the interaction between MoS_2_ and oxidizing gas molecules in the ambient, such as O_2_ and water vapor, and thus increased the electrical outputs of FET devices as observed by the increase in the FET current. Similar FET transfer curves collected after 30, 60, 90 and 120 min indicates the device was more stable than the one without the photoresist but still suffered from the previously observed degradation. Therefore, this experiment series removed the possibility of mechanism (2) of the resistance increasing due to water intercalating between gold and MoS_2_ and confirmed mechanism (3), i.e., water intercalated between conducting channel and the Si/SiO_2_ substrate causing the delamination of MoS_2_ film. This resulting poor contact between MoS_2_ and the dielectric SiO_2_ layer decreased the gating effect onto the conducting channel in FET measurement, yielding the false positive response in detection. In fact, the delamination phenomenon is a commonly used method for isolating CVD-grown MoS_2_ from its growth substrate. Due to the high hydrophobicity of the substrate and MoS_2_, water is used as a medium for a clean, complete lift-off for MoS_2_ as reported elsewhere [24,25].

Even though the as-synthesized MoS_2_ appeared as a continuous film as shown in Figure 1a, it is believed that the film consisted of many single MoS_2_ crystals with distinct grain boundaries. Therefore, further investigation on grain boundaries of the as-grown CVD-MoS_2_ was conducted to understand in more depth the delamination phenomenon that resulted in the instability of MoS_2_-based device. Work function of a similar CVD-SL-MoS_2_ was measured at 4.90 eV in our previous work [8] while the absolute potential of Au^3+^/Au is 5.64 eV. The difference in the work functions creates an intrinsic internal force driving electrons from n-type MoS_2_ to Au^3+^ ions and reduced the ions to Au nanoparticles (NPs). This reduction happens at the reactive sites on MoS_2_’s lattice, which concentrates at its edge and grain boundaries [26,27,28] and, hence, MoS_2_ grain boundaries become evidently detectable due the presence of formation of gold NPs. Figure S3 shows scanning electron microscopic (SEM) images taken at the same location before and after an incubation of MoS_2_ in 5.9 mM AuCl_3_ solution for 10 min. The grain boundaries that were indistinguishable originally became evident due to the formation of Au NPs. This indicates the as-grown continuous MoS_2_ film, in fact, consisted of many coalescing MoS_2_ crystals. These existing large grain boundaries permitted water molecules to severely penetrate through MoS_2_ film and delaminate it from the substrate.

**Figure 4:**
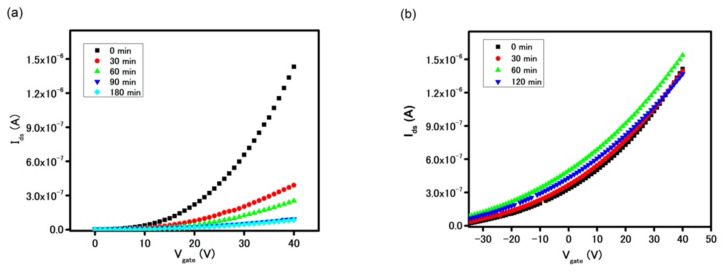
FET characteristics curves of the sensor with (**a**) 10 nm and (**b**) 30 nm Al_2_O_3_ after incubation in DI water in different time ranges.

**Figure 5:**
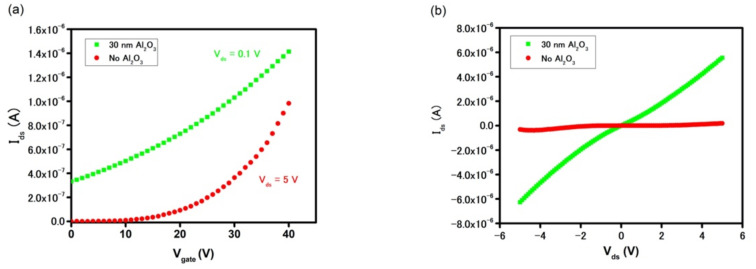
(**a**) FET characteristics curve and (**b**) drain current characteristics curve (V_gate_ = 0 V) of MoS_2_-based device with and without passive Al_2_O_3_ layer.

This finding suggests that a physical passivation layer is required to minimize the intercalation of water molecules through MoS_2_ grain boundaries leading to delamination of MoS_2_ in aqueous solutions. Although a hydrophobic polymeric layer such as S1813 proved to alleviate the degradation of the FET device characteristics, its chemical inertness to surface chemical modification inhibits the subsequent biofunctionalization. On the other hand, thin metal oxide layers, such as hafnium oxide, silicon oxide, and aluminum oxide, are more commonly used as passive layers in logic electronics to protect the underneath material from being oxidized in the ambient [29,30]. In addition, the oxide layer, in presence of ambient and/or water vapor, produced sufficient hydroxyl groups making the device’s surface amenable for biofunctionalization. In this work, the aluminum oxide layer was deposited on the sensors using an ALD method [31]. This deposition process involved two steps, in which the first step provided hydroxyl seeding by casting water vapor on the device‘s surface while the second step was a chemical reaction between -OH groups and TMA to form the Al_2_O_3_ layer. The schematic of MoS_2_-based FETs with a Al_2_O_3_ passive layer is shown in Figure S4. Figure S5 shows the different color contrast of a device before and after Al_2_O_3_ deposition confirming the successful deposition of an oxide layer on the device. The thickness of the Al_2_O_3_ layer on the SiO_2_ substrate was measured at 30 nm by ellipsometer, which can be found in Figure S5c. The oxide layer’s thickness was optimized by measuring the device’s stability after incubation in DI water. Figure 4 demonstrates a device with a 10 nm thick Al_2_O_3_ layer suffered from the degradation. On the other hand, the delamination phenomenon subsided in the device coated with a 30 nm thick oxide layer, as proven by the stable FET transfer curve of the device after 120 min. It is important to highlight that a strong n-doping effect of Al_2_O_3_ onto MoS_2_ was also noticed in the FET and drain current measurements, as seen in Figure 5 [32,33]. Due to its intrinsic semiconducting properties and relatively large work function, CVD-MoS_2_ exhibits low charge mobility and the high contact resistance at MoS_2_/gold interface. However, when the device was passivated by Al_2_O_3_ film, the insignificant current of 3 × 10^−10^ A at V_gate_ = 0 V was increased by three orders of magnitude to 3 × 10^−7^ A, while the field-effect charge mobilities increased by 26-fold, from 14 cm^2^V^−1^s^−1^ to 367 cm^2^V^−1^s^−1^ in the as-grown MoS_2_ and Al_2_O_3_-MoS_2_, respectively. Figure 5a indicates the threshold voltage (V_TH_) of the device also shifted to a more negative threshold voltage (V_TH_) indicating a strong n-doping effect in MoS_2_ by Al_2_O_3_. This interesting observation renders two possible reasons, including the incomplete oxidation of Al_2_O_3_ and the passivation of sulfur vacancy defects. During the Al_2_O_3_ growth, the incomplete reaction between TMA and water vapor resulted in excess positive charged Al^n+^ ions. The residual positive charges in the oxide layer, hence, attracted additional electrons in the MoS_2_ channel and promoted the n-doping effect. This led to the appreciable current found in the Al_2_O_3_ passivated device at V_gate_ = 0 V. On the other hand, as-grown CVD-MoS_2_ exhibited a great number of sulfur vacancies at the boundaries and edges, as observed in the previous experiment with gold chloride solution. These vacancies created deep and localized states trapping electrons and, hence, impaired electron mobility in MoS_2_ [34,35]. However, the presence of the Al_2_O_3_ layer allowed its oxygen atoms to fill sulfur vacancies and, in turn, minimized electrons trapping. Such a healing mechanism has been reported previously using other chemicals such as CO, NO, NO_2_, and other sulfonate groups [35,36]. To the benefit of device electrical performance, this resulting increase in current and charge mobility provided additional electrical measurement method with less power consumption (no gate and lower V_ds_) and simpler fabrication and operation due to the elimination of the gate. Figure 5b highlights such an increase in drain current in the gateless FET device with an Al_2_O_3_ passive layer. At V_gate_ = 0 V, the I_ds_ current is as high as 9.2 × 10^−7^ A at V_ds_ = 1 V and 5.6 × 10^−6^ A at V_ds_ = 5 V (with Al_2_O_3_), as compared to 7.1 × 10^−10^ A and 1.8 × 10^−7^ A (without Al_2_O_3_), respectively.

**Figure 6:**
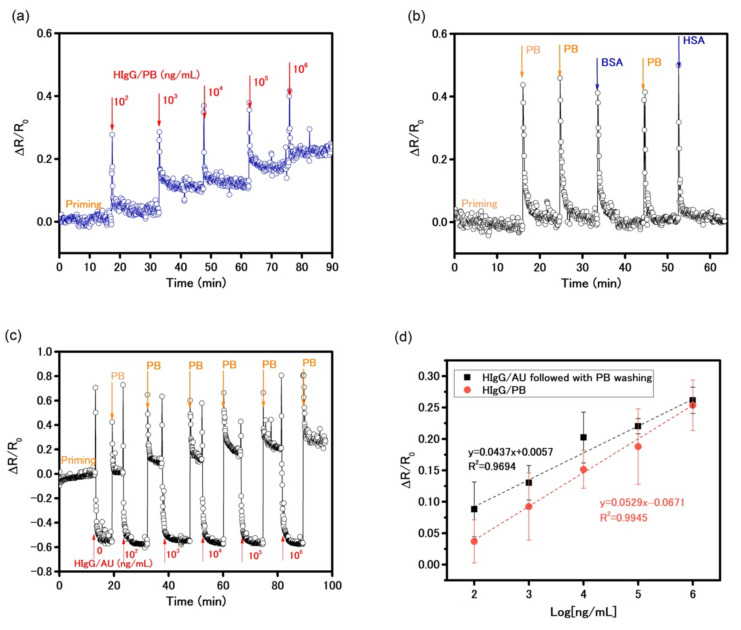
(**a**) Real-time monitoring of the changes in resistance for HIgG sensing and the priming process with 10 mM PB (V_ds_ = 1 V and V_gate_ = 0 V). (**b**) Control sensing experiment with PB solution and high concentration nonspecific antigens of BSA (10^5^ ng/mL) and HSA (10^5^ ng/mL). (**c**) Real-time monitoring of the changes in resistance for HIgG sensing in AU with 10 mM PB wash (V_ds_ = 1 V and V_gate_ = 0 V). (**d**) Calibration curves of Al_2_O_3_ passivated MoS_2_-based sensor for HIgG detections in PB and AU solutions. Data points are average of 3 measurements and error bars represent ±1 SD.

As hydroxyl groups were available on the Al_2_O_3_ layers after ammonium hydroxide treatment, anti-HIgG was covalently functionalized on the device via surface chemical reactions using APTES and glutaraldehyde to construct a biosensor to detect HIgG in PB solution. The device was initially primed with PB solution for stabilization. Then, increasing concentrations of HIgG solution were injected into the cell while the resistance of the sensor was monitored for 15 min. The result in Figure 6a shows the time-dependent change in resistance. It was evident that a spike in the readings was due to the physical injection at each injection point and yet the increase in the reading after stabilization was indeed due to increasing amounts of HIgG. It showed a stabilized signal was achieved in an average of 2 min after each addition of the target solution. It is important to note that the oxide layer, due to the strong n-doping effect, permitted electrical measurement without requiring a back-gate potential that allowed a more facile and low-power mode measurement. The biosensor exhibited good sensitivity by showing clear responses to HIgG solution of concentrations down to 100 ng/mL, as well as high selectivity with non-response to PB solutions, 10^5^ ng/mL of BSA, and 10^5^ ng/mL of HSA solutions, showed in Figure 6b. The calibration curve plotted in Figure 6d shows a linear relationship (y = 0.0529x − 0.0671; R^2^ = 0.9945) between HIgG concentration and the percentage change in resistance within a wide dynamic range from 10^2^ to 10^6^ ng/mL in PB solution. The sensitivity of 0.0529 per Log_10_ (ng/mL) was obtained from the slope of the response (∆R/R_0_) as a function of HIgG concentration, where R_0_ is the resistance of the device established with PB solution in the priming process and ∆R is a resistance change caused by affinity binding of HIgG antigen-antibody interaction. From the noise data of the PB baseline in Figure 6a, root-mean-square value of ∆R/R_0_ noise of ~0.034 was obtained. The limit of detection (LOD), defined as 3 × SD/m (where SD is the standard deviation of the blank and m is the slope of calibration plot), was estimated to be 83 ng/mL. Additionally, the small error bars demonstrate good reproducibility, which assures a reliable consistency in detecting HIgG.

A biosensor that can be used directly with physiological samples is much favored and very demanding. The sensor was further used to detect the HIgG in an artificial urine (AU) matrix (pH = 7.4), which simulated the real-life human body fluids sensing environment [37]. Since the AU has high ionic strength due to a lot of salts, it reduced the Debye length and thereby sensor response. To alleviate this problem, we introduced the washing of the sensor with PB solution to reduce the ionic strength post antibody-antigen binding to recover the responses of MoS_2_-based biosensor. Figure 6c illustrates dynamic response of the biosensor at an applied source-drain voltage of 1 V (with no gate voltage) to 0–10^6^ ng/mL HIgG in AU. After PB priming, the injection of increasing concentrations of the target in AU produced large negative responses, which were opposite to the response obtained in PB (Figure 6a). After washing the test cell by 800 μL of PB, the responses became positive and showed agreement with the responses in PB. The calibration plot of the biosensor to HIgG in AU, shown in Figure 6d, had a linear relationship with the sensitivity (slope) of 0.0437 per Log_10_ (ng/mL), which was in very good agreement with the sensitivity of 0.0529 per Log (ng/mL) in PB. These results present a simple approach for overcoming the effects from organics and salts present in AU and a high degree of accuracy even in a complex real-life sensing environment. Comparison with recent literature results on MoS_2_-based FET biosensors, presented in Table S1, clearly indicates the comparable performance of our MoS_2_ biosensor based on simple chemiresistive principle using modulation of resistance instead of FET principle based on change in transfer characteristic of the device.

## 4. Conclusions

In conclusion, this work presents an extended deliberation for electrical measurement analysis of biosensors. Although the sensitivity aspect of a biosensor is intensively researched, a device’s accuracy, specifically in an event of false positive/negative response, should be equally considered. CVD-grown MoS_2_, due to the nature of its synthesis process, forms a great number of grain boundaries. As a result, a device using such materials is predisposed to undesirable delamination of the material from the substrate. A facile and effective strategy to mitigate this molecularly induced delamination process via physical passivation by atomic layer deposition of the aluminum oxide layer was demonstrated. In addition, the sensors passivated with such oxide layer benefit from a strong n-doping effect allowing another method of detection requiring lower source-drain bias and no gate. These metal oxide-passivated devices not only exhibit a higher physical and chemical stability but also demonstrate high sensitivity and selectivity in biosensing even in a complex real-life sensing environment. This fundamental finding paves the way to further expected improvement of the sensitivity of such biosensors using an optical gating as demonstrated in our previous work [38]. While electrical gating can damage the dielectric substrate by creating pin holes in the layer after repeating applied cycles and require a high range of tens of voltage, photogating is not only lower power consumption and compatible for portable on-chip designs but also has no detrimental effects on the physical device.

## Data Availability

Not applicable.

## Supplementary Materials

The following are available online at https://www.mdpi.com/article/10.3390/bios11120514/s1, Figure S1: Raman spectra of MoS_2_ before and after 30 min incubation in DI water. Figure S2: S1813-MoS_2_-based device (a) schematic and (b) field-effect transistor characteristics after in DI water (pH = 7). Figure S3: SEM image of same location (a) before; (b) after 10 min incubation in 5 mM AuCl_3_ solution. Figure S4: Schematic of MoS_2_-based FETs with Al_2_O_3_ passive layer. Figure S5: Optical image of the sensor (a) before and (b) after Al_2_O_3_ deposition. (c) L1: SiO_2_ and L2: Al_2_O_3_ thickness measured by ellipsometer. Table S1: Performance summary of MoS_2_-based FET biosensors.
