# Supplementary material for: Effect of Al2O3 Passive Layer on Stability and Doping of MoS2 Field-Effect Transistor (FET) Biosensors
Source: Biosensors (Basel). 2021 Dec 13;11(12):514. doi: 10.3390/bios11120514 (PMC8699215; doi:10.3390/bios11120514)
Supplement: Supplementary file 1 [file biosensors-11-00514-s001.zip › biosensors-1433002-supplementary.pdf]

SUPPLEMENTARY INFORMATION

Effect of Al_2O_3 passive layer on stability and doping of MoS_2 Field-Effect Transistor (FET) biosensors

Tung Pham[1#], Ying Chen[1#], Jhoann Lopez[1], Mei Yang[2], Thien-Toan Tran[3], and Ashok Mulchandani[1,4*]

[1]Department of Chemical and Environmental Engineering, University of California Riverside, Riverside, CA 92521, United States

[2]Key Laboratory of Biorheological Science and Technology, Ministry of Education, College of Bioengineering, Chongqing University, Chongqing 400044, PR China

[3]Department of Bioengineering, University of California Riverside, Riverside, CA 92521, United States

[4]Center for Environmental Research and Technology (CE-CERT), University of California Riverside, Riverside, CA 92507, United States

[#]Authors contributed equally

*Corresponding author

Ashok Mulchandani (*adani@engr.ucr.edu*)

W. Ruel Johnson Chair in Environmental Engineering

Distinguished Professor, Chemical & Environmental Engineering

University of California, Riverside

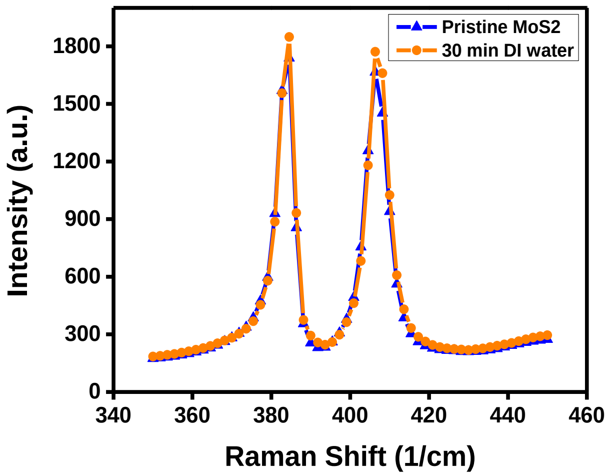

Figure S1. Raman spectra of MoS_2 before and after 30-min incubation in DI water.

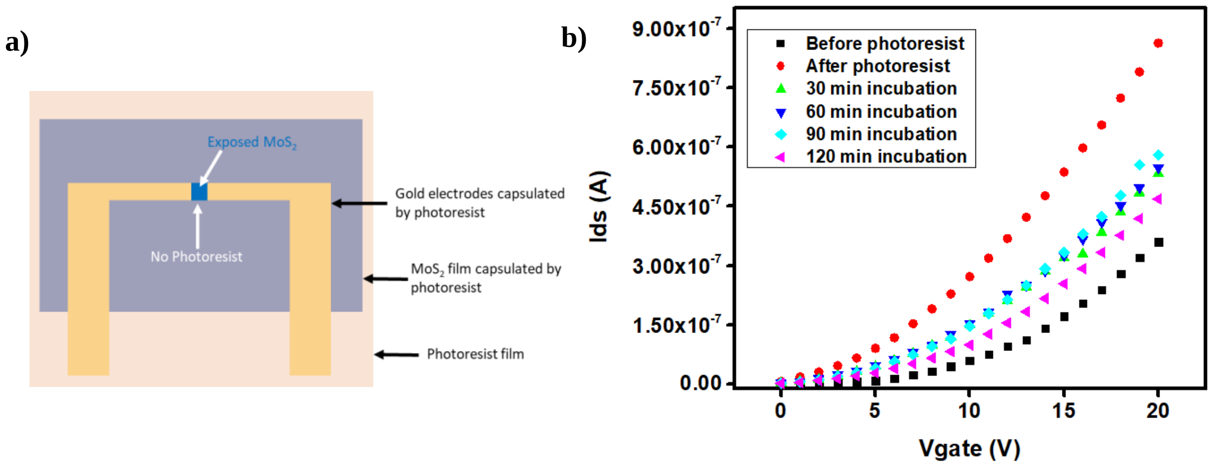

Figure S2. S1813-MoS_2 based device (a) schematic and (b) field-effect transistor characteristics after in DI water (pH=7).

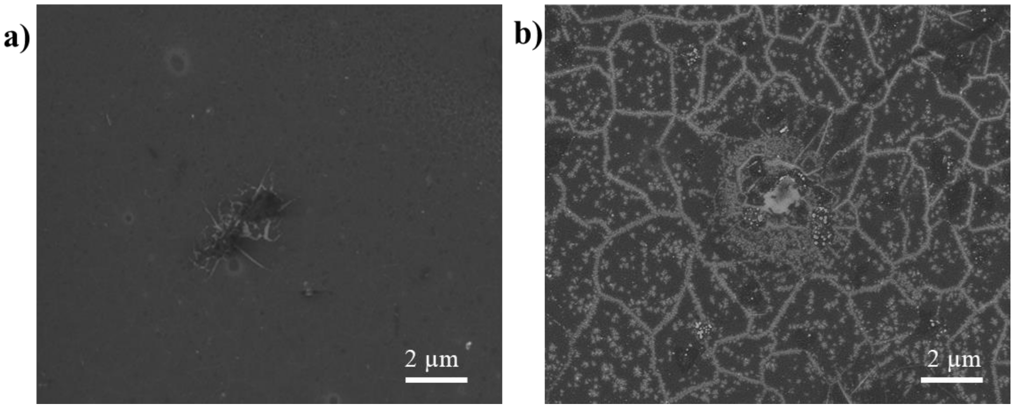

Figure S3. SEM image of same location a) before; b) after 10 min incubation in 5 mM $AuCl_3$ solution.

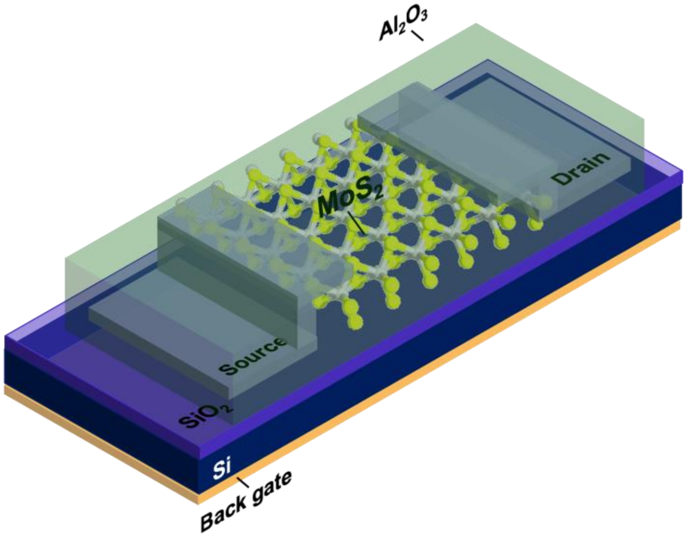

Figure S4. Schematic of MoS_2-based FETs with Al_2O_3 passive layer.

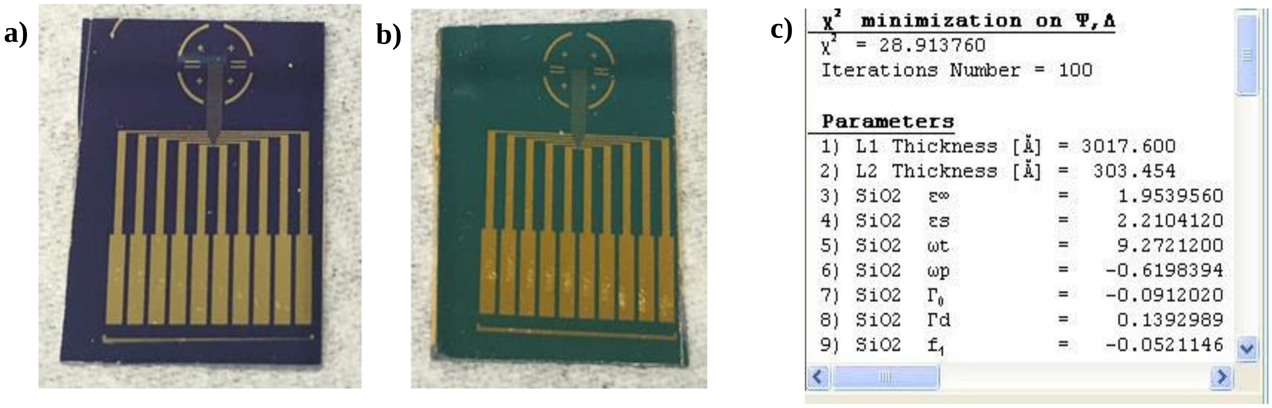

Figure S5. Optical image of the sensor (a) before and (b) after Al_2O_3 deposition (c) L1: SiO_2 and L2: Al_2O_3 thickness measured by ellipsometer.

Table S1. Performance summary of MoS_2-based FET biosensors.

Materials	Target analyte	Concentration range	Sensitivity	LOD	Reference
MoS_2 nanosheets	Prostate-specific antigens (PSA)	10^{-6} ~ 125 ng/mL	0.1295 nA/ng·mL^{-1}	10^{-5} ng/mL	[1]
Exfoliated MoS_2	Glucose	300 nM ~ 30 mM	260.75 mA/mM	300 nM	[2]
CVD MoS_2	Bladder cancer biomarkers NMP22 and cytokeratin 8 (CK8)	10^{-15} ~ 10^{-9} mg/mL	-	0.027 and 0.019 aM	[3]
Commercial MoS_2 nanosheets	C-reactive protein (C-RP)	100 fg/mL ~ 10 ng/mL	176 nA/g·mL^{-1}	8.38 fg/mL	[4]
Printed few-layer-MoS_2	Streptavidin	1 ~ 300 fM	-	1 fM	[5]
Microprinting MoS_2	Tumor necrosis factor-alpha (TNF-α)	60 fM ~ 6 pM	0.14 ± 0.02%/fM	-	[6]
Mechanical exfoliated multilayer MoS_2	PSA	375 fM ~ 3.75 nM	-	-	[7]
Mechanically exfoliated MoS_2	PSA	100 fg/mL ~ 1 ng/mL	-	100 fg/mL	[8]
CVD MoS_2	HIgG	10^2 ~ 10^6 ng/mL (667 pM ~ 6.67 μM)	0.053 per Log (ng/mL)	83 ng/mL	This work

References

1. Kukkar, M.; Tuteja, S.K.; Sharma, A.L.; Kumar, V.; Paul, A.K.; Kim, K.H.; Sabherwal, P.; Deep, A. A New Electrolytic Synthesis Method for Few-Layered MoS2 Nanosheets and Their Robust Biointerfacing with Reduced Antibodies. *ACS Appl. Mater. Interfaces* **2016**, *8*, 16555–16563.

2. Rawat, B.; Mishra, K.K.; Barman, U.; Arora, L.; Pal, D.; Paily, R.P. Two-Dimensional MoS2-Based Electrochemical Biosensor for Highly Selective Detection of Glutathione. *IEEE Sens. J.* **2020**, *20*, 6937–6944.

3. Yang, Y.; Zeng, B.; Li, Y.; Liang, H.; Yang, Y.; Yuan, Q. Construction of MoS2 field effect transistor sensor array for the detection of bladder cancer biomarkers. *Sci. China Chem.* **2020**, *63*, 997–1003.

4. Dalila, N.R.; Arshad, M.K.M.; Gopinath, S.C.B.; Nuzaihan, M.N.M.; Fathil, M.F.M. Molybdenum disulfide—gold nanoparticle nanocomposite in field-effect transistor back-gate for enhanced C-reactive protein detection. *Microchim. Acta* **2020**, *187*, 588.

5. Ryu, B.; Nam, H.; Oh, B.R.; Song, Y.; Chen, P.; Park, Y.; Wan, W.; Kurabayashi, K.; Liang, X. Cyclewise Operation of Printed MoS2 Transistor Biosensors for Rapid Biomolecule Quantification at Femtomolar Levels. *ACS Sensors* **2017**, *2*, 274–281.

6. Nam, H.; Oh, B.R.; Chen, P.; Chen, M.; Wi, S.; Wan, W.; Kurabayashi, K.; Liang, X. Multiple MoS2 transistors for sensing molecule interaction kinetics. *Sci. Rep.* **2015**, *5*, 1–13.

7. Wang, L.; Wang, Y.; Wong, J.I.; Palacios, T.; Kong, J.; Yang, H.Y. Functionalized MoS2 Nanosheet-Based Field-Effect Biosensor for Label-Free Sensitive Detection of Cancer Marker Proteins in Solution. *Small* **2014**, *10*, 1101–1105.

8. Park, H.; Han, G.; Lee, S.W.; Lee, H.; Jeong, S.H.; Naqi, M.; Almutairi, A.; Kim, Y.J.; Lee, J.; Kim, W.J.; et al. Label-Free and Recalibrated Multilayer MoS2 Biosensor for Point-of-Care Diagnostics. *ACS Appl. Mater. Interfaces* **2017**, *9*, 43490–43497.
